# Mechanisms of Efficient Desalination by a Two-Dimensional Porous Nanosheet Prepared via Bottom-Up Assembly of Cucurbit[6]urils

**DOI:** 10.3390/membranes12030252

**Published:** 2022-02-23

**Authors:** Feng Zhou, Jaewoo Lee, Rong Wang, Haibin Su

**Affiliations:** 1 Department of Chemistry, The Hong Kong University of Science and Technology, Hong Kong 999077, China; zhou0128.2012@gmail.com; 2 Department of Polymer-Nano Science and Technology, Jeonbuk National University, 567 Baekje-daero, Deokjin-gu, Jeonju-si 54896, Korea; 3 Department of Bionanotechnology and Bioconvergence Engineering, Jeonbuk National University, 567 Baekje-daero, Deokjin-gu, Jeonju-si 54896, Korea; 4 School of Civil and Environmental Engineering, Nanyang Technological University, Singapore 639798, Singapore; rwang@ntu.edu.sg; 5 Singapore Membrane Technology Center, Nanyang Environment and Water Research Institute, Nanyang Technological University, Singapore 637141, Singapore

**Keywords:** two-dimensional material, cucurbituril, desalination, molecular dynamics simulation, quantum mechanics calculation

## Abstract

Many researchers have examined the desalination performance of various kinds of two-dimensional (2D) porous nanosheets prepared by top-down approaches such as forming pores on the plain based on molecular dynamics (MD) simulations. In contrast, it is rare to find MD simulations addressing the desalination performance of a 2D porous nanosheet prepared by bottom-up approaches. We investigated the desalination performance of a 2D porous nanosheet prepared by the assembly of cucurbit[6]uril (CB[6]) via MD simulation. The model 2D CB[6] nanosheet features CB[6] with the carbonyl-fringed portals of 3.9 Å and the interstitial space filled with hydrophobic linkers and dangling side chains. Our MD simulation demonstrated that the 2D porous CB[6] nanosheet possesses a 70 to 140 times higher water permeance than commercial reverse osmosis membranes while effectively preventing salt passage. The extremely high water permeance and perfect salt rejection stem from not only CB[6]’s nature (hydrophilicity, negative charge, and the right dimension for size exclusion) but also the hydrophobic and tightly filled interstitial space. We also double-checked that the extremely high water permeance was attributable to only CB[6]’s nature, not water leakage, by contrasting it with a 2D nanosheet comprising CB[6]-spermine complexes. Lastly, this paper provides a discussion on a better cucurbituril homologue to prepare a next-generation desalination membrane possessing great potential to such an extent to surpass the 2D porous CB[6] nanosheet based on quantum mechanics calculations.

## 1. Introduction

Two-dimensional (2D) nanomaterials such as graphene oxide or MXenes have received much attention as a compelling alternative for membrane fabrication owing to their unique properties capable of inducing low frictional water flow through the interlayer spacing (or d-spacing) in recent years [1,2]. However, the use of 2D nanomaterials has been restricted mainly to ultrafiltration or molecular separation (i.e., nanofiltration) in many cases due to their larger permeation cutoff than hydrated ions [1,3,4]. For this reason, even the best graphene laminates for desalination have exhibited a much lower performance than in-house polymeric RO membranes (e.g., 4.68–5.54 L m^−2^ h^−1^ bar^−1^ with 98.2–98.9% NaCl rejection in RO using a 2000 NaCl feed solution [5,6,7,8]), although their water permeance was almost 10 times lower than in-house ones. For example, the GO laminate membrane made by spray coating an aqueous solution containing GO and few-layered graphene/deoxycholate exhibited 0.46 L·m^−2^·h^−1^·Bar^−1^ with 85% NaCl rejection in RO using a 2000 NaCl feed solution [9]. As another example, the laminate membrane consisting of chemically converted graphene using tannic acid showed 0.36 L m^−2^ h^−1^ bar^−1^ with 80–92% NaCl rejection in RO using a 500–2000 NaCl feed solution [10]. These results imply that the d-spacing of laminate membranes is too large to separate monovalent ions and too tortuous to allow fast water transport as compared to polymeric RO membranes. As a result, laminate membranes are still too far from practical application in seawater desalination.

In contrast to the laminates, porous nanosheets with sub-nanometer pores have been regarded as the ultimate form to realize the ideal selectivity and outstanding water permeability simultaneously, which was supported by a few simulation studies [11,12,13,14]. For instance, a single-layer graphene nanosheet showed several orders of magnitude higher water permeability than the existing RO membranes while separating ions adequately during MD simulations [11]. Furthermore, the possibility of improving the desalination performance of porous single-layer nanosheets has been reported consistently through MD simulations accompanied by the modification of pore designs or desalination conditions up to recently [15,16,17]. Such porous nanosheets are likely to be fabricated by means of state-of-the-art technologies such as direct focused ion beam (FIB) drilling [18]. However, in reality it is challenging to prepare finely perforated 2D nanomaterial-based membranes with pores small enough to reject ion species with the currently available technologies. To the best of our knowledge, the smallest aperture size that can be formed by direct FIB drilling is about 3 nm [19], which is too large to separate ions from seawater. All things considered, a bottom-up approach could be a more realistic way, as of now, to prepare feasible 2D nanomaterial-based membranes for desalination.

In a bottom-up manner to develop more permeable and selective membranes, it is of importance to carefully choose novel materials for selective water transport over ions. In this context, one needs to note the possibility of designing synthetic water channels as building blocks based on the biological structures or self-assembly of supra-molecules and utilizing them to offer great advantages in terms of the permselectivity of desalination membranes [20,21,22]. Among several synthetic water channels, cucurbituril homologues, which are macrocycles with sub-nanometer pores [23,24,25], can be considered as a promising candidate for selective water transport owing to its well-defined channel structure and carbonyl-fringed portal. However, this material has not yet been explored as a single-layer membrane in spite of its great potential. For example, Baek et al. prepared laminate membranes consisting of 2D cucurbit[6]uril (CB[6]) nanosheets for molecular separation after filling CB[6]’s cavity with guest molecules to fully delaminate the 2D nanosheets [26]. However, their membrane was far from a single-layer membrane with desirable pores for selective water transport due to the preparation method (i.e., laminate membranes and cavities filled with guest molecules). Meanwhile, Cao et al. [27] and Lee et al. [28] have recently demonstrated the great potential of CB[6] for selective water transport in the form of polymeric nanocomposite membranes, but their membranes were also inappropriate for exploring the performance of a single-layer CB[6] membrane.

In this study, we carried out molecular dynamics (MD) simulations to estimate the desalination performance of a 2D porous nanosheet assembled with CB[6]s. A model 2D porous CB[6] nanosheet was assembled by referring to the structure reported in the previous study [29] except for filling the cavity with a guest molecule. Our MD simulations revealed that the model porous nanosheet possesses great potential as a next-generation desalination membrane by showing superfast water transport and perfect salt rejection. Additionally, we double-checked that the extremely high water permeance of the 2D CB[6] nanosheet does not result from leakage but CB[6]’s nature. This fact was evidenced by contrasting it with a 2D nanosheet consisting of host–guest complexes (i.e., cavities filled with guest molecules). Lastly, quantum mechanics (QM) calculations were performed to predict the best candidate among cucurbituril homologues to construct a single-layer porous nanosheet for desalination.

## 2. Molecular Dynamics (MD) Simulation and Quantum Mechanics (QM) Calculation

All simulations were carried out using the GROMACS 4.5.3 suite of programs [30]. The CB[6] and water molecules were described using the amber03 force field [31] and the tip5p water model [32], respectively. All simulations were conducted under periodic boundary conditions at constant temperature and pressure (NPT). The initial coordinates of the small molecules were made from Gaussview [33]. The SPC/E, PPC, and TIP4P [34], and BSV, CC, DC, SPC/E, and TIP4P [35] models were reported as failing to adequately represent the experimental O···O radial distribution function. The TIP3P and SPC exhibit particularly poor agreement, while the TIP4P, SPC/E, and PPC show better agreement. However, the recent models TIP4P-FQ and increasingly used TIP5P [32] provide further improvement [36] at a higher computational cost. In this paper, we used TIP5P to model water for MD simulation studies.

As for QM calculations, all species used in the energy scan regarding hydrated sodium ions approaching the cavity center were entirely optimized by the density functional theory (DFT) at the M06-2X/6-31g(d) level [37,38]. All computations were performed with Gaussian 09 package [39]. The convergence thresholds were set as follows: 10^−3^ a.u. for the gradient, 10^−3^ a.u. for the displacement, and 10^−6^ a.u. for the energy, respectively. We utilized the polarizable continuum model (PCM) to model the water environment for the QM calculation.

## 3. Results and Discussion

### 3.1. MD Simulations to Determine the Desalination Performance of the 2D Porous CB[6] Nanosheet

**Figure 1:**
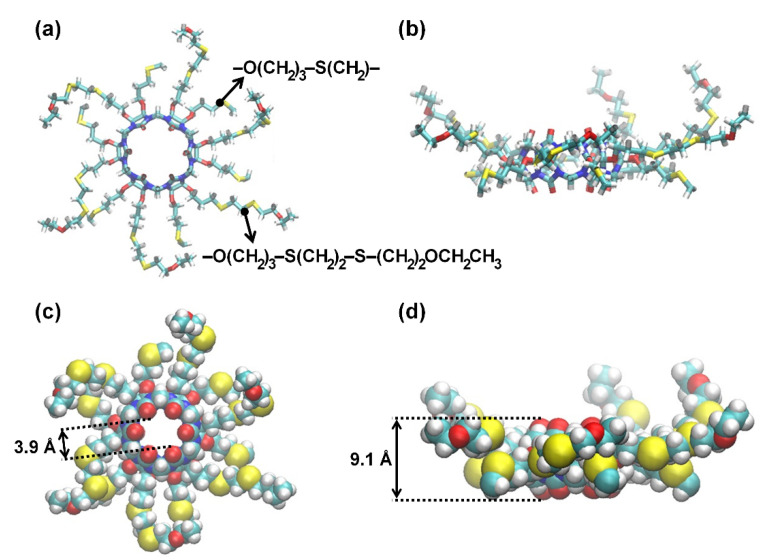
Top (**a**,**c**) and side (**b**,**d**) views of the modified CB[6] with six thioether bridges (−O(CH_2_)_3_−S(CH_2_)−) and dangling arms (−O(CH_2_)_3_−S(CH_2_)_2_S−(CH_2_)_2_OCH_2_CH_3_). −O(CH_2_)_3_−S(CH_2_)− corresponds to a symmetric half of the linker. (**a**,**b**) do not include van der Waals spheres, whereas (**c**,**d**) do. Color scheme: N, blue; C, cyan; O, red; H, white; S, yellow.

**Figure 2:**
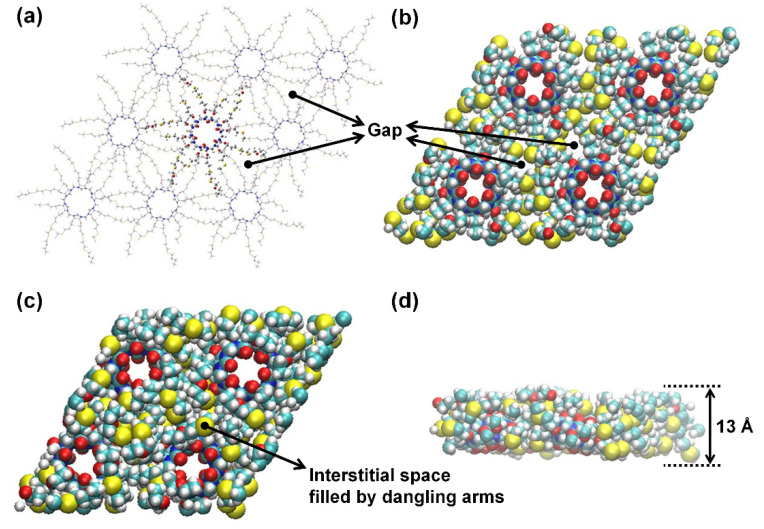
Top view of the 2D CB[6] nanosheet with (**a**) sticks and (**b**) spheres models at 0 ns. (**c**) Top view and (**d**) side view of the 2D CB[6] nanosheet at 10 ns. The gaps between the neighboring CB[6] units observed at 0 ns were determined to be filled by the dangling arms, which were intended to form a thicker and denser hydrophobic region. Once the dangling arms agglomerated to fill the empty interstitial space, the hydrophobic region (about 13 Å) became thicker than the height (9.1 Å) of CB[6]. Color scheme: N, blue; C, cyan; O, red; H, white; S, yellow.

The previous study demonstrated that CB[6] can be used to improve the permselectivity of thin-film nanocomposite reverse osmosis membranes thanks to the selective water transport characteristics of CB[6] [28]. However, the ideal future scenario would be to construct a single-layer film by seamlessly assembling CB[6]s on top of a porous support to make full use of CB[6]’s performance. Thus, we carried out MD simulations using the GROMACS 4.5.3 package [30] to predict the desalination performance of a 2D porous CB[6] nanosheet. A 2 × 2 supercell was used to realize an infinite 2D CB[6] nanosheet (Figure 1 and Figure 2), which comprises four CB[6] units, the thioether bridges to link the neighboring CB[6] units, −O(CH_2_)_3_−S(CH_2_)_2_S−(CH_2_)_3_O−, and the dangling side chains −O(CH_2_)_3_−S(CH_2_)_2_S−(CH_2_)_2_OCH_2_CH_3_, filling the interstitial space between CB[6] units, as reported previously [29]. The initial system configuration was subject to periodic boundary conditions and consisted of a box measuring approximately 39 Å (x) × 39 Å (y) × 102 Å (z). We placed 1409 water molecules on the saltwater side along with 60 NaCl ions, and 1465 water molecules were placed on the freshwater side. The salt water had a concentration of 2.37 M, which has an osmotic pressure of 115 bar. In our simulation, the osmotic pressure drives water molecules to transport across the CB[6] nanosheet. Thus, the high concentrated salt water was used to simulate a higher applied pressure than the osmotic pressure of seawater of about 0.6 M, and the water permeability coefficient is evaluated by dividing the water flux by the osmotic pressure.

**Figure 3:**
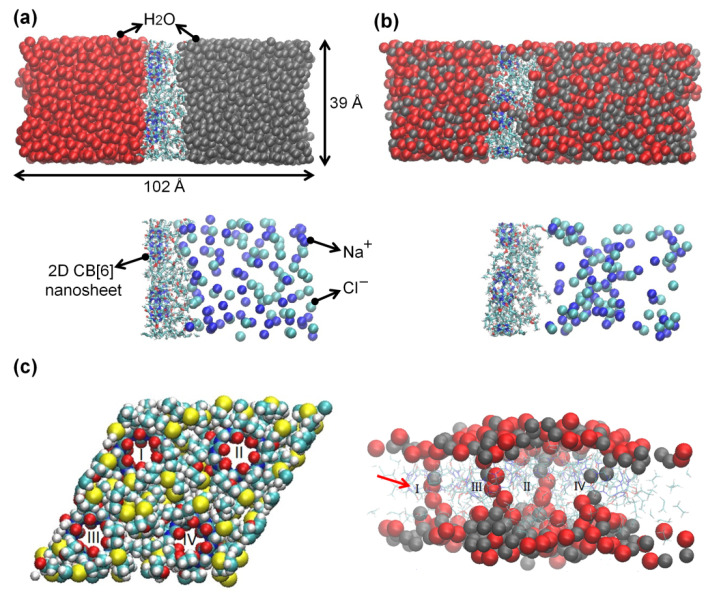
Simulation system consists of the 2D CB[6] nanosheet, pure water (red sphere, left), and a 2.37 M NaCl solution (gray sphere, right) at (**a**) 0 and (**b**) 100 ns. Water molecules and NaCl ions of salt water are presented separately for clarity. (**c**) Top view of the 2D CB[6] nanosheet (left) and side view of the simulation system including water molecules within 5 Å away from the 2D CB[6] nanosheet at 100 ns (right).

Figure 3a,b illustrate the setup of our simulation on the water flow across the 2D CB[6] nanosheet. During the 100 ns simulation, 350 water molecules migrated across the 2D CB[6] nanosheet. The water transport rate was calculated to be 7.6 × 10^6^ water molecules unit^−1^ sec^−1^ bar^−1^, translating into 212 L m^−2^ h^−1^ bar^−1^. This intrinsic water permeability is about 70 to 140 times higher than commercial RO membranes. More importantly, such excellent water permeability was achieved without any salt passage. NaCl ions can hardly diffuse through not only the CB[6] portal due to the remarkable electrostatic repulsion [28] but also the inter-CB[6] space because of the hydrophobic nature of the linker and the effective space filled by side chains. The dynamic behavior of water molecules in the CB[6] nanosheet is presented in Figure 3c, which demonstrates that water molecules are predominantly passing through the CB[6] cavity instead of the hydrophobic interstitial space. To be specific, water molecules randomly distributed at 0 ns were arranged and then migrated along four CB[6] channels (red arrow) at 100 ns. This implies that water molecules mostly permeate through the CB[6] cavity, whereas the hydrophobic interstitial space is not preferred for water permeation when the CB[6] portal is open.

### 3.2. Investigation of the Possibility of Water Leakage through the Interstitial Space

We also attempted to determine whether the high water permeance of the 2D CB[6] nanosheet results from CB[6]’s features, not leakage through the inter-CB[6] space. To test the possibility of water leakage, we prepared another 2D nanosheet by replacing four CB[6] units with four CB[6]-spermine complexes. According to the previous study [28], the host–guest complex consisting of CB[6] and fully protonated spermine was identified to have a much higher binding energy (−231.9 kcal mol^−1^) than CB[6]·H2O (−14.02 kcal mol^−1^). This fact suggests that water molecules cannot replace the bound spermine and thereby permeate through the CB[6]-spermine complex from the point of view of energetics. Accordingly, if a significant net flow of water is observed in the MD simulation using the 2D CB[6]-spermine nanosheet, it indicates water leakage through the inter-CB[6] space. Additionally, the degree of water leakage helps us where the extremely high water permeance comes between CB[6]’s nature and the water leakage.

To clarify where the extremely high water permeance of the 2D porous CB[6] nanosheet comes from, we carried out a 100 ns MD simulation using the 2D CB[6]-spermine nanosheet. A 2 × 2 supercell was used to realize the infinite 2D CB[6]-spermine nanosheet, which consists of four CB[6]-spermine complexes, the thioether bridges to link the neighboring CB[6]-spermine complexes, −O(CH_2_)_3_−S(CH_2_)_2_S−(CH_2_)_3_O−, and the dangling side chains, −O(CH_2_)_3_−S(CH_2_)_2_S−(CH_2_)_2_OCH_2_CH_3_, filling the interstitial space between CB[6]-spermine complexes. The initial system configuration was subject to periodic boundary conditions and consisted of a box measuring approximately 39 Å(x) × 39 Å(y) × 126 Å(z). We used 2880 water molecules for both the fresh and salt water; 44 sodium and 60 chloride ions were placed on the saltwater side.

**Figure 4:**
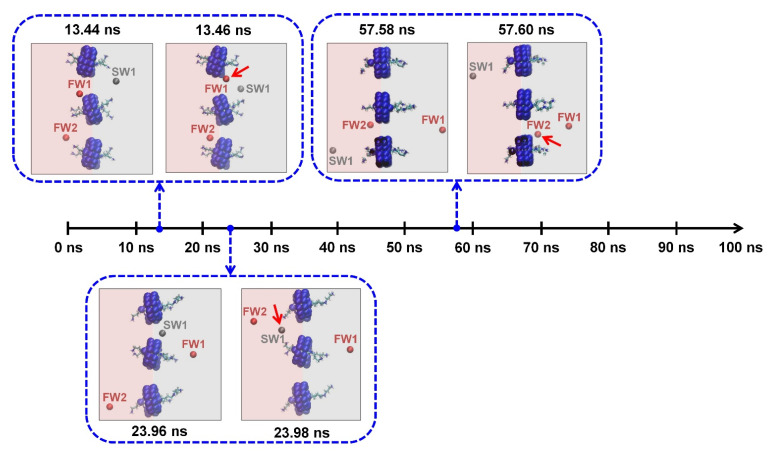
Six frames show water passage across the interstitial space of the 2D CB[6]-spermine nanosheet for 100 ns. The frames represent the three water molecules moving across the interstitial space. The FW1 and FW2 signify two water molecules on the freshwater side, while the SW1 means a water molecule on the saltwater side. The red and grey highlights present the freshwater and saltwater side, respectively. The red arrows indicate the water molecules that moved to the other side. Meanwhile, thioether bridges, dangling arms, NaCl ions, and the other water molecules on the freshwater (red, left) and saltwater (gray, right) sides are not shown for clarity.

The MD simulation shows that no water molecules passed through the CB[6]-spermine complex as expected. Meanwhile, only three water molecules crossed the interstitial space for 100 ns, as shown in Figure 4 and Figure S1. In detail, two water molecules (red spheres) crossed from the fresh water to the salt water, while one water molecule (grey sphere) crossed from the salt water to the fresh water. This result means that the 2D CB[6]-spermine nanosheet reveals the net flow of water corresponding to only one water molecule. The net flow of water through the 2D CB[6]-spermine nanosheet is negligible, even allowing for the fact that the CB[6] cavities were blocked, considering that it was 350 water molecules in the case of the 2D porous CB[6] nanosheet. Accordingly, it is clear from this discussion that the extremely high water permeance of the 2D porous CB[6] nanosheet stems from CB[6]’s nature.

### 3.3. QM Calculations to Weigh Up the Best Candidate among Cucurbituril Homologues

The previous study demonstrated that CB[6] possesses selective water transport characteristics stemming from the carbonyl-fringed portal of 3.9 Å [28]. However, it does not necessarily mean that CB[6] is the best among cucurbituril homologues to make desalination membranes. Accordingly, we tried to determine which is better between CB[6] and CB[7] as a building block for desalination membranes. CB[5] and CB[8] were excluded from consideration because CB[5] has a smaller portal (2.4 Å [25]) than water molecule (2.75 Å), while CB[8] has a larger portal (6.9 Å [25]) than a hydrated chloride ion (6.6 Å). First, we compared the binding energies between one water molecule and the two cucurbituril homologues to weigh up which one is more desirable to afford fast water transport. Here, a lower binding energy equals faster water transport through cucurbituril homologues. CB[6]·H_2_O and CB[7]·H_2_O (Figure S2) showed the binding energies of −14.9 and −1.27 kcal mol^−1^, respectively. This result implies that CB[7] can be more favorable than CB[6] for fast water transport through the cavity owing to the lower binding energy.

**Figure 5:**
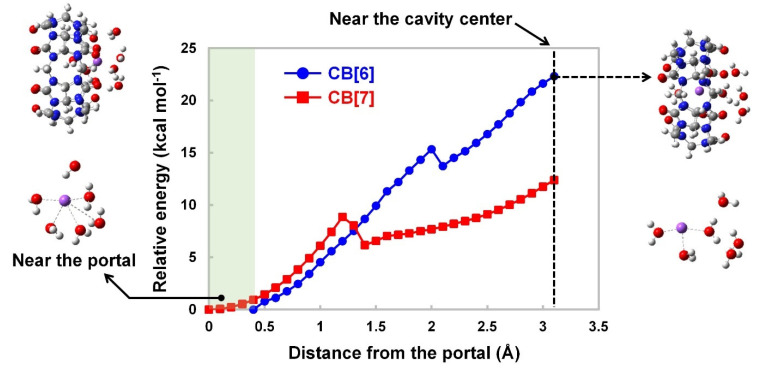
The CB[6]-ion and CB[7]-ion complexes’ relative energies as a function of the distance between a hydrated sodium ion and the portal. The first water shell was used to calculate the energy barrier of a hydrated sodium ion. The molecular structure of a hydrated sodium ion outside CB[6] was described on the left side, while the right illustration shows the structure in the center of the cavity (color scheme: C, grey; N, blue; H, white; O, red; Na, purple).

However, too large of a portal can backfire from the perspective of preventing salt passage. With this in mind, we performed QM calculations using CB[6], CB[7], and a hydrated sodium ion. According to our calculations, as a hydrated sodium ion approached the center of the CB[6] and CB[7] cavities, the relative energies increased by 22.3 and 12.4 kcal mol^−1^, respectively (Figure 5). The increased relative energies are assumed to be associated with geometrical deformation that a hydrated sodium ion would go through in the cavity, as reported previously [28]. More importantly, CB[7] also exhibits a certain level of energy barrier imposed by the cavity while a hydrated sodium ion approaches the cavity center, which suggests that CB[7] is also likely to prevent salt passage. Overall, it is worth trying to examine whether CB[7] is a more desirable building block to prepare a 2D porous nanosheet for desalination in that it could promote fast water transport due to the low binding energy of CB[7]·H_2_O while inhibiting salt passage because of the energy barrier imposed by the CB[7] cavity.

## 4. Conclusions

Our simulation revealed that a single layer of the defect-free 2D porous CB[6] nanosheet is likely to produce fresh water at a rate that is two orders of magnitude faster than commercial reverse osmosis membranes while retaining ions. The extremely high water permeance was found to arise from the carbonyl-fringed portal of 3.9 Å, which was double-checked by negligible water leakage even through the interstitial space between CB[6]-spermine complexes. Lastly, the potential of CB[7] as a building block for desalination membranes was also identified by the low binding energy of CB[7]·H_2_O and the energy barrier imposed by the CB[7] cavity. We expect that this study can play a catalytic role in the development of 2D porous nanosheet for desalination through bottom-up assembly.

## Data Availability

Not applicable.

## Supplementary Materials

The following are available online at https://www.mdpi.com/article/10.3390/membranes12030252/s1, Figure S1: simulation system consisting of the 2D nanosheet with four CB[6]-spermine complexes, Figure S2: optimized structures of CB[6] and CB[7] with one water molecule.
