# Supplementary material for: Mechanisms of Efficient Desalination by a Two-Dimensional Porous Nanosheet Prepared via Bottom-Up Assembly of Cucurbit[6]urils
Source: Membranes (Basel). 2022 Feb 23;12(3):252. doi: 10.3390/membranes12030252 (PMC8954457; doi:10.3390/membranes12030252)
Supplement: Supplementary file 1 [file membranes-12-00252-s001.zip › membranes-1593607-supplementary.pdf]

Supplementary Information

Mechanisms of efficient desalination by a two-dimensional porous nanosheet prepared *via* bottom-up assembly of cucurbit[6]urils

Feng Zhou [1,†], Jaewoo Lee [2,3,†,*], Rong Wang [4,5], Haibin Su [1,*]

[1] Department of Chemistry, The Hong Kong University of Science and Technology, Hong Kong 999077, China

[2] Department of Polymer-Nano Science and Technology, Jeonbuk National University, 567 Baekje-daero, Deokjin-gu, Jeonju-si 54896, Korea

[3] Department of Bionanotechnology and Bioconvergence Engineering, Jeonbuk National University, 567 Baekje-daero, Deokjin-gu, Jeonju-si 54896, Korea

[4] School of Civil and Environmental Engineering, Nanyang Technological University, 639798, Singapore

[5] Singapore Membrane Technology Center, Nanyang Environment and Water Research Institute, Nanyang Technological University, 637141, Singapore

*Corresponding author (J.L.): jaewoolee@jbnu.ac.kr

*Corresponding author (H.S.): haibinsu@ust.hk

[†] These authors contributed equally to this work.

KEYWORDS. Two-dimensional material, Cucurbituril, Desalination, Molecular dynamics simulation, Quantum mechanics calculation

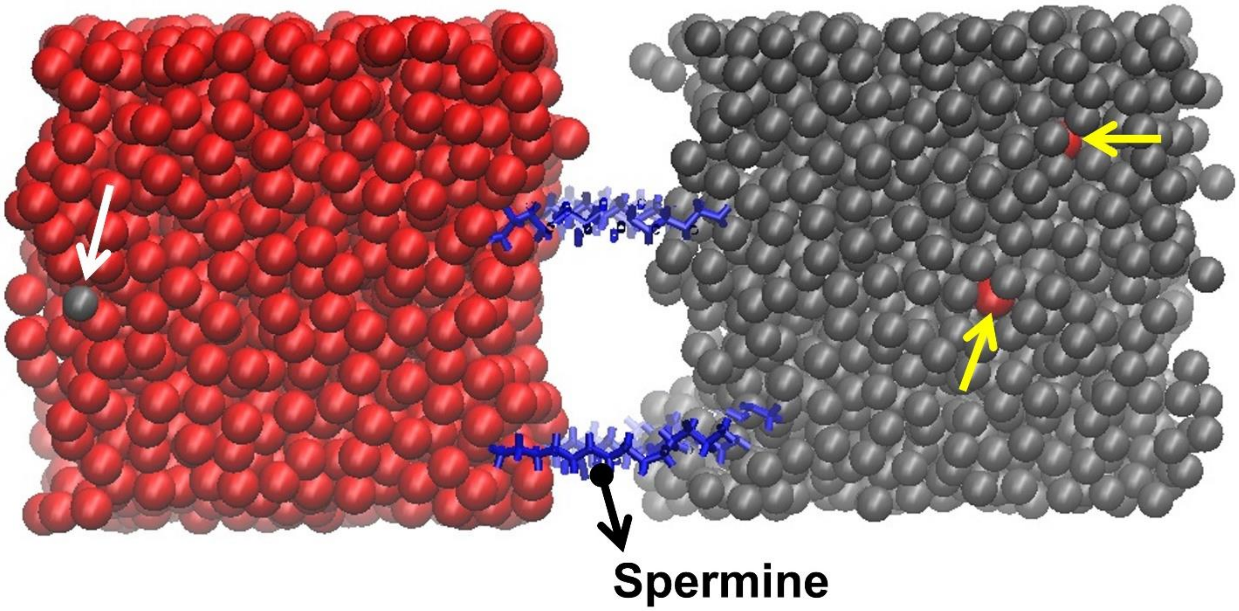

Figure S1. Simulation system consisting of the 2D nanosheet with four CB[6]-spermine complexes, 2880 water molecules, 44 sodium, and 60 chloride ions at 100 ns. The thioether bridges, dangling arms, CB[6], and NaCl ions are not represented for clarity. Red and gray spheres are indicative of water molecules on the freshwater and saltwater sides, respectively. The yellow arrows show two water molecules that moved from the fresh water to salt water, while the white arrow exhibits one water molecule that moved from the salt water to fresh water.

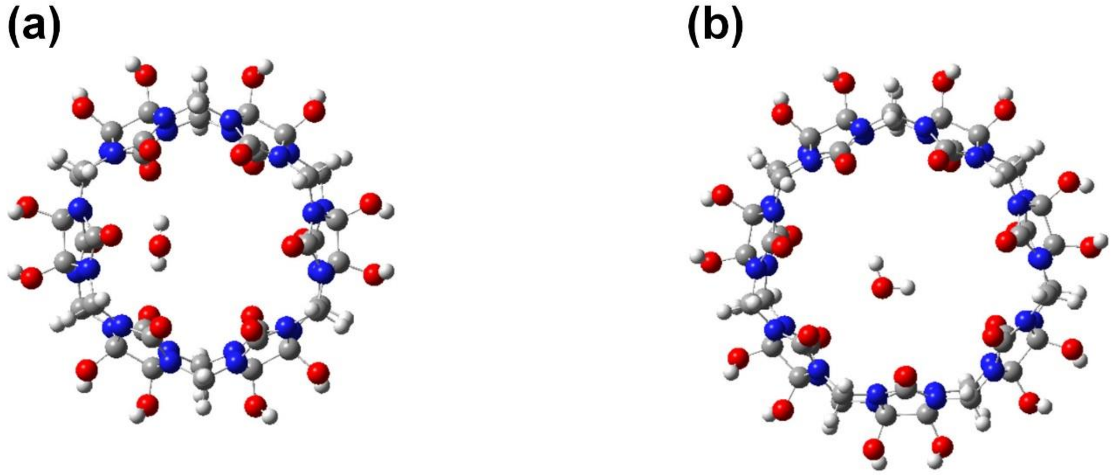

Figure S2. Optimized structures of (a) CB[6] and (b) CB[7] with one water molecule (color scheme: C, grey; N, blue; H, white; O, red). Gaussview was used to generate initial molecular structures [1], which were optimized with the Gaussian 09 package unless stated otherwise [2].

References

[1] R. Dennington, T. Keith, J. Millam, GaussView, version 5, (2009).

[2] M. Frisch, G. Trucks, H. Schlegel, G. Scuseria, M. Robb, J. Cheeseman, G. Scalmani, V. Barone, B. Mennucci, G. Petersson, Gaussian 09, revision E. 01 (2009) Gaussian Inc, Wallingford, CT See Supporting Information for full citation.
